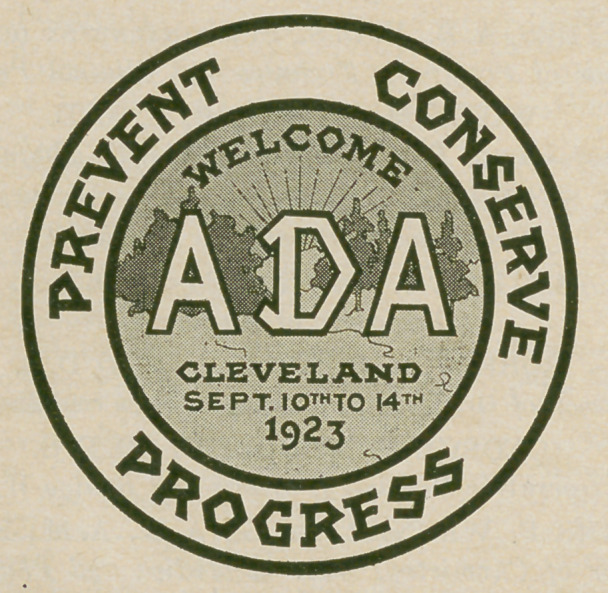# Baccalaureate Address

**Published:** 1923-06

**Authors:** Edwin Godby

**Affiliations:** 1923, Ohio College of Dental Surgery


### BACCALAUREATE ADDRESS BY GRADUATING SENIOR

To 1923 Graduating Class of O. C. D. S.

---

BY DR. EDWIN GODBY, 1923, OHIO COLLEGE OF DENTAL SURGERY

---

I have been selected to address you at this final meeting of the class of 1923, a meeting which brings with it a combined feeling of joy and sadness—joy, because we have reached the goal for which we have been so long striving; sadness, because of the severing of long and intimate companionships.

Four years ago we came to the Ohio College of Dental Surgery from our scattered and various homes, occupations, high schools and universities as strangers to each other and to an untried field of endeavor, but we part to-night as true and sincere friends with one common object and ambition. This assemblage to-night will transfer us from the roll of a student to the place of that honored profession of Dental Surgery. From now on our duties and environments will be much different. Much more will be demanded of us to successfully solve the problems we will meet daily. We must be skilled diagnosticians, and, as such, be familiar on one hand with mechanics, on the other with physiology and pathology; we must have a high degree of technical skill in addition to our general knowledge of medicine and surgery. Our work will also carry us into the field of chemistry, bacteriology, metallurgy, physics, electricity and art. Without an artistic sense we will fall far short of modern standards. Our technical skill may be compared to that demanded to that of the rhinologist and surgeon, inasmuch as he is at all times dealing with human tissue.

In the scheme of dental education we must realize that the art or practice is daily combined with theoretical learning. In other words, the student of dentistry serves his interneship during his four years' course for his degree, and when he receives the degree of Doctor of Dental Surgery as we do to-night, he is competent to enter at once upon practice and serve the public in a satisfactory and skilful manner. We trust our friends in the audience will remember this fact and not hesitate to leave the older practitioners for the new.

The profession of dentistry is a progressive one, ever discovering new truths, and penetrating the mystic shrines of hidden facts, dispelling the age of darkness and changing ignorance into philosophy. We should be ever mindful of the careful study and unremitting toil of our predecessors and let the results of their investigations stand as monuments, and always remember that their results make it easier for us and our patients.

It is true in dentistry as in general medicine that each embrace a field too wide for the ordinary mind to grasp, and of necessity has been divided into specialties, each with its great possibilities and problems.

Do not allow yourselves to believe the practice of dentistry is one of untold delights, a paradise of eternal spring, for many are the handicaps we must face. One of the greatest is that of being a young dentist. It we can gain an early forgiveness for this one sin, we will be several rungs up the ladder of success, but we must not let our many difficulties discourage us, but always remember the lily does not break into full bloom in one morning, but with the warmth of many spring mornings the bud swells and the green deepens and tint is added to tint, and beauty to beauty, until it stands in sovereign glory of perfect bloom.

Professors of the faculty, to you has been given the task of impressing directly upon our minds those truths that shall ever develop the truest manhood of each nature, and implanting in each brain and heart the germ of knowledge whose perfect growth shall form lives of success; how well you have discharged this responsibility the present but faintly knows, the future alone can tell how successfully you have labored in our behalf. We tremble when we leave you, for here we have relied upon your wisdom, your guidance; here we have sought council and assistance from you who have always been willing and able to bestow it. Now we launch our little craft away from the shipyard, out of the harbor we must battle with the waves where there shall be none to guide or assist. Our own eyes must watch the compass and scan the chart, our own hands must man the rudder. If ever hours of dark defeat and failure should come, bitterly will we rue the neglect with which we have met, and when we have made a successful voyage and weigh anchor in a safe harbor we will think of you and say, "That to your wisdom and instruction we owe all." It now becomes my duty on the part of the class to bid you farewell. Your many acts of kindness and words of wisdom have gained our most profound respect and challenge our greatest admiration, now that words will not express the deep emotion which swells in our breasts, we can only say that we thank you. No matter where we shall go, or how long we shall abide, there will always remain in our memory a place for you and our time-honored alma mater.

Fellow classmates, this day will forever be engraved upon our memory, we are assembled for the last time to take part in an occasion which marks an epoch in our lives that time can not erase. Sad we are at parting, yet our hearts are glad, for after years of toil and patient waiting we shall receive that coveted degree of Doctor of Dental Surgery. This is not a time for heavy hearts or for tears, but for joy unspeakable. It is a day of high hopes and expectations, and the advice that falls from the older lips should be carefully weighed lest they chill the ardor of a generous enthusiasm or stay the all-conquering faith of youth that moves the universe.

## Figures and Tables

**Figure f1:**